# Effects of sliding techniques on lower limb biomechanics and muscle synergy during curling delivery: a focus on joint kinetics and muscle coordination

**DOI:** 10.3389/fnhum.2025.1587118

**Published:** 2025-06-27

**Authors:** Lijun Hua, Yulong Zhu, Gengchao Bi, Ming Zhu, Yuzi Diao

**Affiliations:** ^1^School of Sports Education, Harbin Sport University, Harbin, China; ^2^Graduate School, Harbin Sport University, Harbin, China; ^3^Heilongjiang Research Institute of Sports Science, Harbin, China

**Keywords:** curling delivery, sliding methods, curling athletes, lower limb joints, muscle coordination

## Abstract

**Background:**

Different curling delivery techniques, such as full-foot contact (FFC), outward-toed full-foot contact (OTFFC), and toe contact (TC), impose distinct biomechanical demands and neuromuscular control challenges on the sliding leg. However, current research on the specific differences among these techniques in terms of multi-joint coordination and muscle synergy is limited. This study investigates the effects of these three propulsion techniques on the lower limb joint mechanics and muscle synergy patterns of the left sliding leg during curling delivery in curling athletes.

**Methods:**

Kinematic and electromyographic (EMG) data from eight key sliding leg muscles (rectus femoris, vastus medialis, vastus lateralis, semimembranosus, biceps femoris, gastrocnemius, lateral gastrocnemius, tibialis anterior) were recorded from 16 male professional curlers using 3D motion capture and wireless EMG during FFC, OTFFC, and TC techniques. Muscle synergies were extracted via non-negative matrix factorization (NMF), and joint mechanics via inverse dynamics. Differences were assessed using repeated measures ANOVA.

**Results:**

The TC significantly altered hip and knee joint mechanics, notably increasing peak hip abduction, knee flexion, and associated torques (all *p* < 0.01), while also showing greater subtalar joint abduction than OTFFC (*p* = 0.001). Conversely, OTFFC elicited greater knee adduction angles and ankle dorsiflexion torques (*p* < 0.01); FFC compromised subtalar joint stability (*p* < 0.01). Hip and knee adduction torques were generally highest in OTFFC and lowest in TC (*p* < 0.001). TC demonstrated substantially higher knee flexion/extension torques (32–41%, *p* ≤ 0.002). Three distinct muscle synergy patterns were identified: Synergy 1 (hip-knee dominant) showed increased rectus femoris contribution in TC, while Synergy 2 (ankle-foot dominant) exhibited earlier gastrocnemius activation in OTFFC (*p* < 0.05 for synergy findings).

**Conclusion:**

In conclusion, the TC predominantly relies on the coordinated activation of muscle groups responsible for hip abduction, knee flexion/extension, and external rotation. Conversely, the OTFFC emphasizes the coordinated effort of muscle groups surrounding the ankle and subtalar joints. In terms of injury prevention, the concentrated use of the hip and knee joints in the TC technique suggests that targeted strengthening and stability training for these areas should be implemented to prevent potential overuse injuries.

## 1 Introduction

As a precision-based sport involving dynamic balance and tactical decision-making, curling has been increasingly studied for its biomechanical demands (Murata, 2022). The delivery technique is a determinant of competitive success, requiring precise coordination to project stones into target areas with sub-meter accuracy (Yoo et al., 2012; Wu et al., 2024). The sliding phase constitutes the biomechanical foundation of delivery, as it governs energy transfer efficiency and body stability. Specifically, the left sliding foot (in right-handed athletes) serves as the primary load-bearing structure, enabling force transmission and motion control through stable support (Engebretsen et al., 2010). Based on the ice-contact patterns of the sliding foot, curling delivery techniques can be categorized into three types: full-foot contact, out-toed full-foot contact, and toe contact sliding. These different propulsion techniques may not only affect the joint mechanics of the left lower limb but also trigger different neuromuscular coordination strategies. These strategies, in turn, can influence the accuracy and stability of the curling delivery. Quantifying the effects of sliding techniques on joint kinetics and muscle synergy in the left leg thus provides critical insights for optimizing athletic performance and injury prevention.

Biomechanical analysis of the sliding phase is essential for understanding how FFC/OTFFC/TC techniques regulate motion stability and injury risk. From a kinematic chain perspective, Kraemer demonstrated a strong correlation between trunk inclination and hip flexion angles during sliding, indicating that hip joint kinematics of the left leg are primarily modulated by trunk posture. The weaker association between femoral and acetabular movements suggests dominant contributions from hamstring and gluteus medius activation for dynamic stability (Kraemer, 2009). Kinetic analysis reveals that TC sliding generates significantly higher knee joint forces (approximately double those of FFC), with increased ground reaction force moment arms potentially compromising tibiofemoral stability (Robertson et al., 2017). Performance comparisons between elite and recreational athletes show that elites achieve larger ankle dorsiflexion ranges and greater anterior center of mass displacement, correlating with more efficient quadriceps-hamstring co-activation patterns (Yoo et al., 2012). Three-dimensional motion capture data further indicate that FFC/OTFFC/TC techniques induce individualized joint torque modulation strategies, emphasizing the need for customized neuromuscular control protocols based on plantar contact patterns (Harms, 2000).

Although existing studies have explored the curling delivery motion from the perspectives of kinematics and kinetics, analyzed the joint kinematic and kinetic parameters during the delivery process of curling athletes, and pointed out the potential changes in joint loading associated with toe contact propulsion techniques (Harms, 2000; Yoo et al., 2012; Robertson et al., 2017), most of these studies have been limited to descriptive analyses or isolated joint mechanics. Currently, there is a lack of comprehensive research on how different propulsion techniques systematically affect the neuromuscular control strategies of the left sliding leg in athletes, thereby ensuring sliding stability and delivery accuracy. Despite curling being a low-impact sport, its specific technical movements and postures are still prone to overuse and static injuries (Engebretsen et al., 2010; Berry et al., 2013). However, few studies have examined the association between these injuries and specific propulsion techniques from the perspective of neuromuscular coordination dysfunction. Therefore, the uniqueness of this study lies in using muscle synergy theory as the core framework to systematically compare and analyze how the lower limb muscles are organized and activated by the nervous system in these curling propulsion techniques. This not only helps athletes optimize their propulsion methods, enhance delivery accuracy and stability, and thus improve their competitive level, but also provides key references for injury prevention in curling athletes and promotes the development of the sport.

The muscle synergy theory posits that the central nervous system (CNS) executes complex motor tasks by activating a set of preset, functionally related synergistic modules or elements. These modules coordinate muscle contractions in specific patterns to accomplish movement, rather than independently controlling each muscle (Singh et al., 2018; Cheung and Seki, 2021). Acting as the “fundamental components” of motor control, these synergistic modules produce a variety of adaptive motor behaviors through different combinations and temporal activations. Their goal is to reduce the redundancy of brain control while ensuring movement stability and efficiency. In recent years, driven by advances in neuroscience and sports science, the muscle synergy theory has been widely applied in sports such as badminton and basketball shooting. Studies have demonstrated that muscle synergy is crucial for movement accuracy and stability (Matsunaga and Oshikawa, 2022; Pan et al., 2024).

In curling delivery, which demands high levels of body coordination, stability, and precise lower limb control, athletes must adapt to ice conditions and tactical requirements by using different propulsion techniques. The muscle synergy theory provides a powerful analytical framework for understanding these complex muscle coordination strategies. However, research in this area remains limited. To further understand how these strategies achieve efficient movement in the multi-joint, multi-muscle human system, computer simulation and biomechanical modeling are essential. The human musculoskeletal system—composed of bones, joints, muscles, and other tissues—can be effectively analyzed in a non-invasive manner using musculoskeletal modeling theory, which continues to evolve (Zang et al., 2023). In the field of winter sports, musculoskeletal models have been successfully applied to sports such as alpine skiing, ski jumping, and snowboarding (Senner et al., 2015; Huang Y. et al., 2023; Gao et al., 2024), demonstrating significant potential. Nevertheless, systematic research on how different propulsion techniques in curling affect muscle synergy patterns is still scarce.

The purpose of this study was to reveal the mechanisms by which different sliding methods affected the biomechanical characteristics of the left lower limb joints and muscle coordination patterns during curling delivery through systematic experimental design and data analysis. To this end, we employed advanced motion capture systems and force sensors to precisely collect kinematic and kinetic data of the left lower limb joints of curling athletes under different sliding methods. Simultaneously, we used surface electromyography (EMG) technology to analyze muscle coordination patterns. We extracted and analyzed the corresponding muscle synergy modules in accordance with muscle synergy theory. By conducting a comprehensive analysis of these data, we explored in depth how different sliding methods impact the biomechanical characteristics of the left lower limb joints and the coordination between muscles. This provided a scientific basis for optimizing the sliding methods of curling athletes and improving delivery performance. Based on this, the following hypotheses are proposed: (1) Different sliding methods had significant differences in the joint angles and moments of the hip, knee, and ankle joints of the lower limbs. (2) There were significant differences in muscle contribution values within each synergy under different sliding methods, and significant differences in muscle coordination activation patterns at different stages of the movement cycle.

## 2 Materials and methods

### 2.1 Participant

A total of 16 male curling athletes from the Harbin Institute of Physical Education participated in this study. All participants had approximately 3 years of curling training experience, with an average age of 22.10 ± 2.38 years, height of 1.839 ± 0.049 m, and body weight of 77.95 ± 4.43 kg. Participants were required to major in curling training. Those meeting the following conditions were excluded: (1) individuals injured during the testing period; (2) individuals who had sustained lower limb injuries within the past 3 months, as these could potentially interfere with the study results. Additionally, to eliminate potential interference from differences between left and right sliding legs, athletes who used their right leg as the supporting leg during delivery were also excluded.

Before the study began, it was strictly reviewed and approved by the Ethics Committee of the Harbin Institute of Physical Education (Ethics Approval No.: 2024012). Prior to testing, researchers thoroughly informed all participants about the study’s purpose, design, and potential risks and benefits to ensure full understanding. All participants voluntarily signed an informed consent form.

### 2.2 Experimental process

This study utilized 13 high-speed infrared cameras from the Qualisys 600 series V5 (Sweden), featuring a 5-megapixel resolution and a sampling frequency of 100 Hz. Additionally, a 16-channel wireless surface electromyography (EMG) system, Trigona by DELSYS (USA), was used, with a sampling frequency of 2000 Hz. Kinematic and surface electromyographic data during the entire curling delivery motion were synchronously collected using Qualisys Tracker Manager (QTM) software.

Before the experiment, participants should be briefed on the test content, standards, and precautions. They should also perform warm-up activities. To minimize the influence of upper limb movements on the lower limbs during testing, participants are required to maintain standardized and stable upper limb movements. Additionally, to ensure consistent ice surface conditions, the ice was inspected and treated before each experimental session in accordance with standard curling rink maintenance protocols. These protocols aimed to maintain uniform ice thickness and smoothness, thereby minimizing variations in surface friction that could affect biomechanical measurements. The experimental venue’s temperature should be controlled within an appropriate range: the ice surface temperature should be maintained between −7°C and −4°C, and the air temperature 1.5 meters above the ice should typically be kept around 10°C to minimize the impact of temperature on athletes’ physical condition and performance.

**Figure 1:**
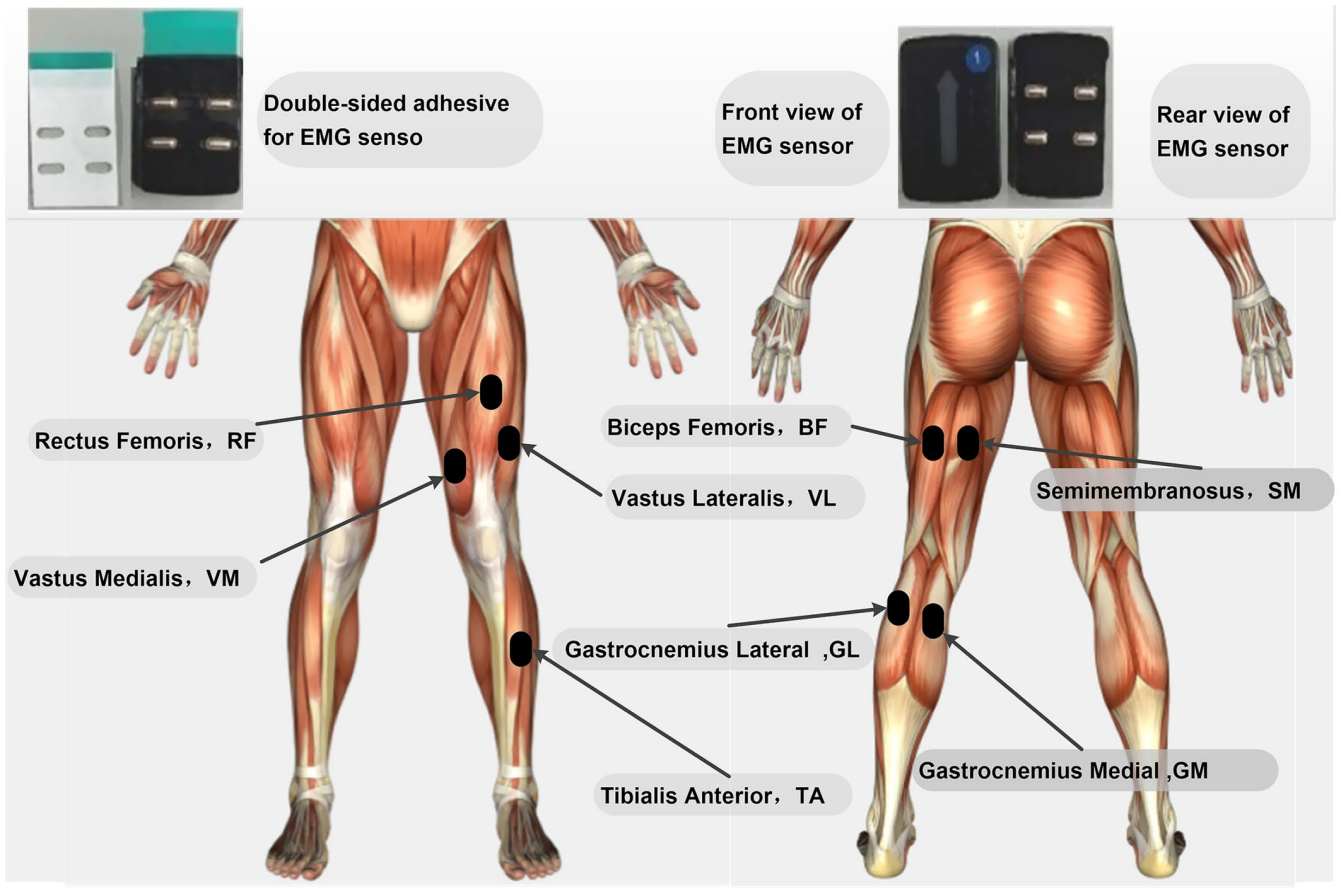
Specific locations for surface EMG sensor placement.

After preparation, EMG sensors were attached to the rectus femoris, vastus medialis, vastus lateralis, semimembranosus, biceps femoris, medial gastrocnemius, lateral gastrocnemius, and tibialis anterior of the left lower limb (specific locations are shown in Figure 1). Before attachment, the target muscle areas were thoroughly cleaned with alcohol swabs to remove oils and dirt from the skin surface. Any hair that might obstruct sensor contact was shaved off to ensure optimal sensor-skin contact. The sensors were then affixed using double-sided tape along the direction of the muscle fibers at the belly of the target muscles, as illustrated in Figure 1. The placement of all electrodes was conducted in accordance with the guidelines recommended by the SENIAM (Surface Electromyography for the Non-Invasive Assessment of Muscles) project. After sensor placement, activated Trigno surface EMG sensors were positioned along the muscle fibers on the corresponding muscle bellies of the participant’s left lower limb. Reflective markers were then placed according to the Rajagopal model to accurately capture whole-body kinematic data. The primary markers were precisely affixed to key anatomical bony landmarks on both sides of the subject’s body or along the midline. These markers included: the spinous process of the seventh cervical vertebra (C7), the midpoint of the clavicle (CLAV), the bilateral anterior superior iliac spines (R/L ASI) and posterior superior iliac spines (R/L PSI), as well as the bilateral acromial points (R/L ACR), anterior shoulder points (R/L ASH), and posterior shoulder points (R/L PSH). Additionally, there were three markers on each upper arm (R/L UA1-3), the lateral epicondyles of the humerus (R/L LEL) and medial epicondyles of the humerus (R/L MEL), the radius marker on the forearm (R/L FA superior) and the ulna marker (R/L FA radius). For the lower limbs, the markers included three points on each femur (R/L TH1-3), the lateral femoral condyles (R/L LFC) and medial femoral condyles (R/L MFC), three points on each tibia (R/L TB1-3), the lateral malleoli (R/L LMAL) and medial malleoli (R/L MMAL), as well as the calcaneal points (R/L CAL), toe points (R/L TOE), and fifth metatarsal points (R/L MT5). All markers were affixed by the same experienced experimenter to ensure consistency in positioning and data accuracy across different subjects and testing conditions. Before formal data collection, the firmness of all markers and their visibility within the camera field of view were carefully checked.

The procedure for testing curling throws on ice is as follows: Static Posture Collection: Participants stood with their feet shoulder-width apart and arms extended sideways for 3 s on the ice to collect static data. Dynamic Action Collection: Under the experimenter’s command, participants sequentially performed full-foot contact, out-toed foot contact, and toe contact movements while dynamic kinematic and EMG signals were collected. After completing each action, the experimenter recorded the data and checked whether any reflective markers or sensors had detached, ensuring the integrity and accuracy of the EMG signal data before concluding the standard test. A 60-s rest interval was provided between trials. Testing Conclusion: After all participants completed the test, reflective markers, and EMG sensors were removed. Raw data were saved, and equipment was returned to its designated place, marking the end of the experiment.

### 2.3 The Rajagopal model, three delivery techniques, and phases of movement

The Rajagopal model,^1^ developed by Rajagopal et al. (2016), includes the skeletal geometry of the entire body, kinematics with 37 degrees of freedom at the joints, Hill-type models driving 80 muscle-tendon units in the lower limbs, and 17 ideal torque actuators controlling the upper body (Rajagopal et al., 2016; Lai et al., 2017).

**Figure 2:**
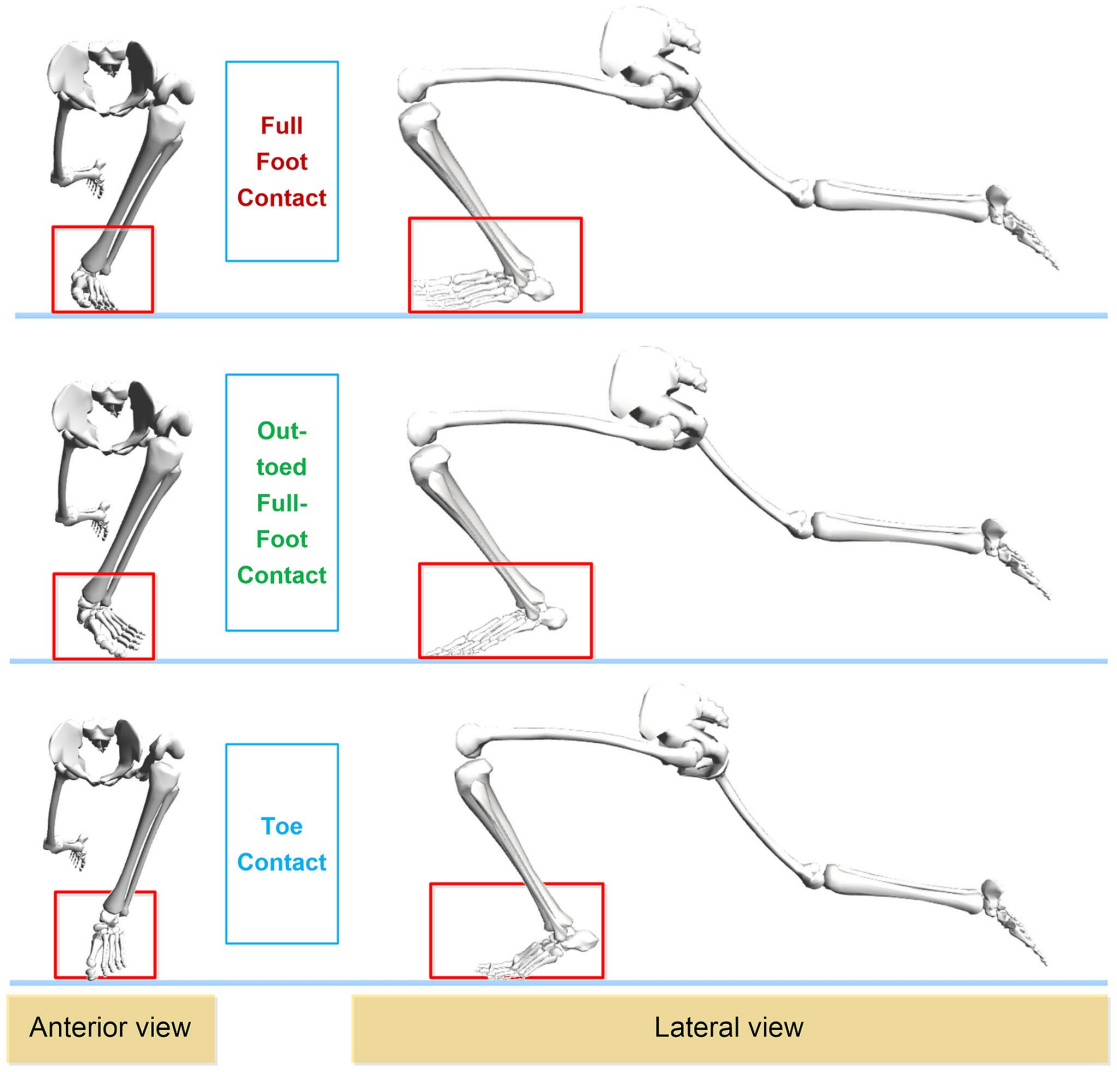
Frontal and lateral diagrams of the three sliding methods used by curling athletes during delivery.

This study employs the sophisticated Rajagopal musculoskeletal model to specifically focus on the lower limb joint biomechanics and muscle synergy patterns of curling athletes. Therefore, only the lower limb bones are shown in Figure 2 to highlight the differences among the three gliding methods. As illustrated in Figure 2, the front and side views of the three gliding methods during the final stage of the delivery action (gliding phase) are presented. It is clearly visible that the athlete’s left leg serves as the supporting leg, with the left foot contacting the ice surface in three distinct ways: full-foot contact (FFC), out-toed full-foot contact (OTFFC), and toe contact (TC). These gliding methods are highlighted in red rectangular boxes in the figure.

Full-Foot Contact: During gliding, the entire sole of the foot makes even contact with the ice surface, with all parts of the sole supporting the body weight. The toes point straight forward, maintaining a neutral position. Out-toed Full-Foot Contact: In this method, the outer edge of the foot contacts the ice surface more than the inner side, resulting in an everted foot position. Compared to Full-Foot Contact, the foot is turned outward. Toe Contact: In this method, the forefoot, particularly the metatarsal head, primarily contacts the ice surface, while the heel has minimal or no contact with the ice surface.

**Figure 3:**
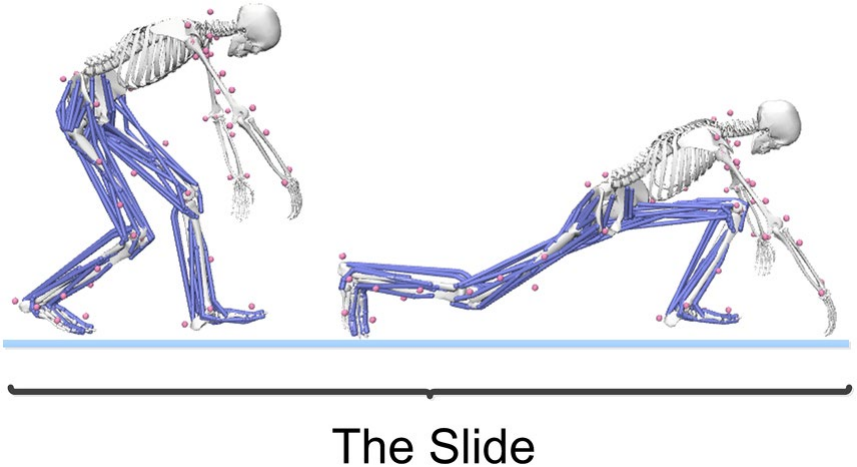
The gliding phase of the curling delivery action.

The curling delivery action is typically divided into several phases: forward push, pull-back, gliding, release (and rotation), and follow-through (Berry et al., 2013). This study focuses on the gliding phase, as illustrated in Figure 3. After completing the pull-back motion, the athlete generates force by pushing off the hack with the right leg while flexing the left knee and placing the left foot on the ice to form a support, adopting a “kneeling glide” posture. At this point, the athlete holds the curling stone with the right hand and the broom with the left hand to ensure a smooth glide.

### 2.4 Data processing of skeletal model and extraction of muscle synergies

First, the captured motion data were processed using low-pass filters at 10 Hz and 100 Hz to smooth the kinematic and kinetic parameters, respectively. Next, the Rajagopal model was used for motion simulation. The OpenSim simulation process consists of the following steps: Scaling: The dynamic model and static kinematic data were scaled to match the subject’s dimensions, resulting in a scaled model. Inverse Kinematics: Dynamic kinematic data were applied to the scaled model to calculate joint angles and other kinematic parameters. Inverse Dynamics: Kinematic data were applied to the scaled model for inverse dynamics analysis (Seth et al., 2011, 2018).

Next, the raw surface electromyography (sEMG) signals collected during the experiment are preprocessed, which mainly includes the following steps: ① Filtering: The frequency range is set to 20–500 Hz to remove low-frequency motion artifacts (such as noise caused by skin movement). Then, a 60 Hz notch filter is used to eliminate power frequency interference. ② Full-wave rectification: The filtered sEMG signals are fully rectified, converting the signal values to absolute values (Bi et al., 2024). ③ Low-pass smoothing and linear envelope extraction: A fourth-order Butterworth low-pass filter (cut-off frequency of 6 Hz) is used for smoothing to generate the linear envelope. The preprocessed electromyographic data matrix is then obtained for muscle synergy extraction (Chvatal and Ting, 2013).

Finally, this study uses the Non-negative Matrix Factorization (NMF) algorithm is used to extract muscle synergies (Lee and Seung, 1999), whose mathematical expression is:


EMG=W×H+ϵ

Where: $\mathrm{EMG}\in R^{m\times n}$ The preprocessed electromyography (EMG) data matrix, where m represents the number of muscle channels and n represents the number of time points. $W\in R^{m\times k}$: The muscle weight matrix, reflecting the contribution ratio of each muscle in the synergy pattern. $H\in R^{k\times n}$: The activation pattern matrix, depicting the intensity changes of the synergy pattern over time. *ϵ*: The residual term. k: The preset number of synergies (Shuman et al., 2017; Turpin et al., 2021).

The algorithm was implemented using the nnmf function in MATLAB R2023a, with the following key parameter settings: Initialization method: Non-negative Double Singular Value Decomposition (NNDSVD) based on Singular Value Decomposition (SVD) (Qiao, 2015). Optimization algorithm: Multiplicative Update rule. Iteration settings: The maximum number of iterations was set to 1,000, and the convergence tolerance was set to 1 × 10–6 (Hosseinzadeh Aghdam and Daryaie Zanjani, 2021). Repetition validation: Each dataset was independently run 10 times, and the solution with the smallest reconstruction error was selected. To ensure the biological plausibility and reproducibility of the results, the following secondary validations were performed: Variance Accounted For (VAF) screening: Only decomposition results with VAF greater than 85% were retained. The calculation formula is:


$$\mathrm{VAF}=1-\frac{∥\mathrm{EMG}-\mathrm{WH}{∥}_{F}^{2}}{∥\mathrm{EMG}{∥}_{F}^{2}}$$

Where $∥{∥}_{F}^{2}$ enotes the Frobenius norm. Muscle Weight Consistency Test: The stability of the weights from multiple decompositions was evaluated by calculating the intraclass correlation coefficient (ICC). When ICC(3,k) is greater than 0.75, it is considered to have high consistency, while muscle channels with ICC less than 0.5 will be excluded.

Next, normalization is performed on the synergy parameters. Muscle weight normalization refers to the process of normalizing each synergy pattern individually to eliminate amplitude differences. The specific formula is:


$$W_{\mathrm{norm}}^{\left(i\right)}=\frac{W^{\left(i\right)}}{\max(W^{\left(i\right)})}$$

where $W^{\left(i\right)}$is the original weight vector of the i-th synergy.

Activation pattern time alignment refers to using the starting point of the gliding action as a reference and aligning the activation curves across trials through Dynamic Time Warping (DTW) to address the issue of temporal jitter (Izakian et al., 2015). For the identification of synergy patterns, K-means clustering (based on Euclidean distance) is used to group individual synergy patterns, and the clustering quality is assessed using the Silhouette Score (Paparrizos and Gravano, 2017).

### 2.5 Key indicators

The key indicators of this study include the kinematic parameters and joint torque metrics of the hip, knee, and ankle joints under three different gliding methods. These indicators provide an intuitive reflection of how different gliding methods affect the biomechanical characteristics of the lower limb joints. Additionally, this study examines muscle synergy-related indicators from the pull-back phase to the gliding phase, specifically focusing on the number of muscle synergies, the contribution weights of each muscle within the synergy modules, and the curve changes of the movement primitives. By analyzing these indicators, we can gain a deeper understanding of how different gliding methods influence muscle synergy patterns.

### 2.6 Statistical analysis

IBM SPSS Statistics 24 was used to perform descriptive statistical analyses on the lower limb joint angles and joint torques corresponding to the three sets of motion data. Results are presented as mean ± standard deviation. To identify significant differences among groups, a one-way analysis of variance (ANOVA) was conducted. Prior to ANOVA, the data were tested for normality and homogeneity of variance. Multiple comparisons were performed only when the ANOVA results were statistically significant: the Bonferroni method was applied for homogeneous variances, while Tamhane’s T2 method was used for heterogeneous variances, with a significance level set at 0.05 for both. Additionally, the effect size η^2^ was calculated to evaluate the practical significance of the findings comprehensively.

For the one-dimensional curve data of movement primitives in muscle synergies, the F-test in SPM1d was utilized to analyze differences among the three one-dimensional curves across the motion interval. A two-tailed test with a significance level of 0.05 was employed to examine statistical differences in continuous data (Pataky et al., 2016). All SPM1d statistical analyses were performed in MATLAB 2023a.

## 3 Results

### 3.1 Kinematic characteristics

**Figure 4:**
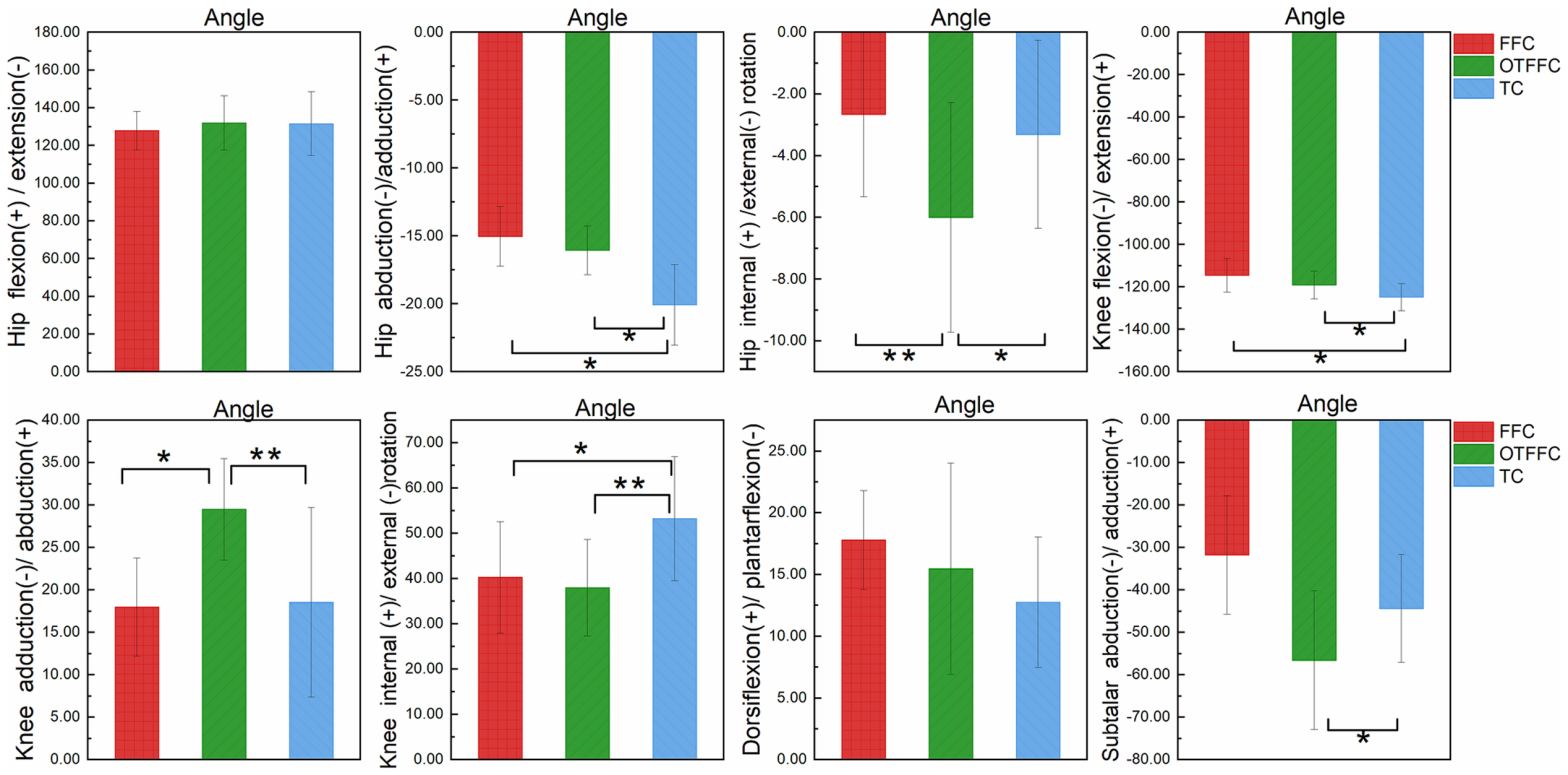
Impact of propulsion techniques on lower limb joint angles during curling delivery. “*” denotes a significance level of *p* <0.05, and “**” denotes a significance level of *p* <0.01.

The results of the one-way ANOVA on the lower limb kinematic indicators under different gliding methods shown in Table 1 and Figure 4 indicate significant differences in joint angles. Specifically: For the hip joint, the abduction angle during the Toe Contact (TC) gliding method is significantly greater than that of FFC and Out-toed OTFFC (*p* = 0.01). Meanwhile, the external rotation angle of the hip joint during OTFFC is significantly greater than that of FFC (*p* = 0.003) and TC (*p* = 0.014). The knee joint angles exhibit multidimensional differences: TC shows the largest flexion angle compared to FFC (*p* = 0.01) and OTFFC (*p* = 0.017). Additionally, the internal rotation angle of the knee joint during TC is significantly higher than that of OTFFC (*p* = 0.003) and FFC (*p* = 0.01). Notably, the adduction angle of the knee joint during OTFFC is significantly greater than that of FFC (*p* = 0.01) and TC (*p* = 0.003). Moreover, the subtalar joint abduction angle during TC gliding is significantly smaller than that of OTFFC (*p* = 0.009). These findings demonstrate that different gliding methods have distinct specificities in their effects on the kinematic parameters of each joint.

**Table 1:** Effects of three gliding methods on the joint angles of the left lower limb during the gliding phase of the delivery action (*n* = 16) (Mean ± SD).

Measure (angle)	FFC	OTFFC	TC	*F*	*p*	*η* ^2^
Hip joint flexion (+)/extension (−)	127.78 ± 10.24	131.91 ± 14.41	131.51 ± 16.98	0.46	0.631	0.02
Hip joint abduction (−)/adduction (+)	−15.03 ± 2.20^c^	−16.05 ± 1.80^c^	−20.08 ± 2.97^ab^	22.67	0.001	0.47
Hip joint internal rotation (+)/external rotation (−)	−2.66 ± 2.67^b^	−6.00 ± 3.72^ac^	−3.31 ± 3.04^b^	5.61	0.006	0.18
Knee joint flexion (−)/extension (+)	−114.59 ± 7.88^c^	−119.17 ± 6.53^c^	−124.87 ± 6.35^ab^	9.87	0.001	0.28
Knee joint adduction (−)/abduction (+)	17.96 ± 5.79^b^	29.49 ± 5.97^ac^	18.52 ± 11.17^b^	11.77	0.001	0.32
Knee joint internal rotation (+)/external rotation (−)	40.27 ± 12.33^c^	37.97 ± 10.64^c^	53.24 ± 13.7^ab^	8.07	0.001	0.24
Dorsiflexion (+)/plantarflexion (−)	17.78 ± 4.02	15.46 ± 8.55	12.75 ± 5.2	2.92	0.063	0.11
Subtalar joint abduction (−)/adduction (+)	−31.76 ± 13.94b^c^	−56.56 ± 16.30^a^	−44.37 ± 12.72^a^	13.36	0.001	0.34

Significant differences between groups are indicated by letters a-c. “a” denotes significant differences compared to the Full-Foot Contact group, “b” denotes significant differences compared to the Out-toed Full-Foot Contact group, and “c” denotes significant differences compared to the Toe Contact group. The values of the effect size index *η*^2^ are defined as follows: 0.01 indicates a small effect, 0.06 indicates a medium effect, and 0.14 indicates a large effect.

### 3.2 Joint kinetic characteristics

**Figure 5:**
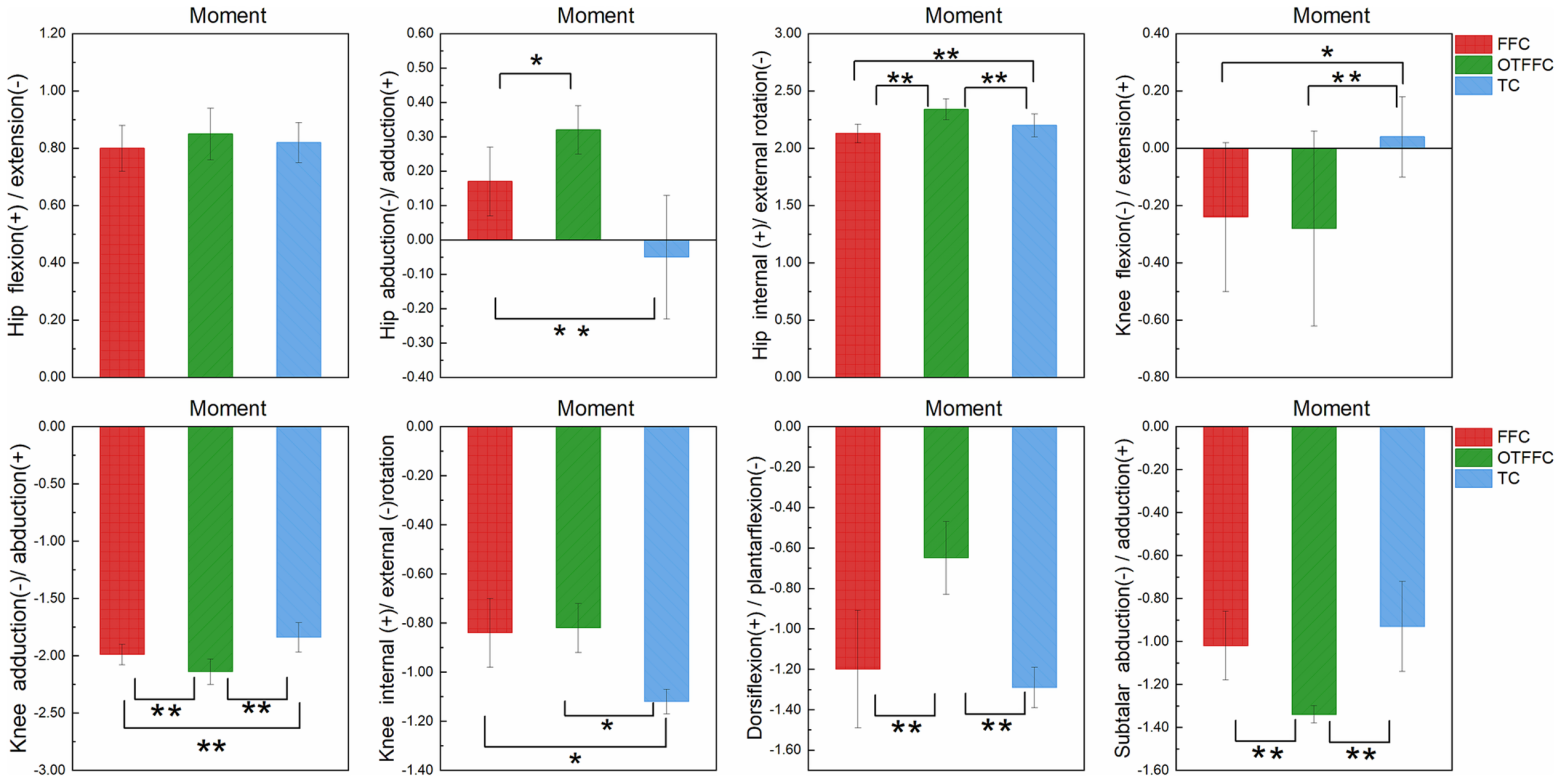
Impact of propulsion techniques on lower limb joint moments during curling delivery. “*” denotes a significance level of *p* <0.05, and “**” denotes a significance level of *p* <0.01.

As shown in Table 2 and Figure 5, the one-way ANOVA on the lower limb joint torque indicators shows that the gliding method has a significant impact on the torque parameters of each joint. The specific patterns of differences are as follows: For the hip joint dynamic adduction torque, there is a gradient relationship of OTFFC group (*p* = 0.001) > FFC group > TC group (*p* < 0.01). For the internal rotation torque, there is a hierarchical difference of OTFFC group > TC group > FFC group (OTFFC vs. FFC/TC: *p* < 0.01; TC vs. FFC: *p* < 0.01). Knee joint kinetic flexion-extension moment values: The TC group was significantly lower than the OTFFC group (*p* = 0.002) and the FFC group (*p* = 0.001); for the adduction torque, there is a step-like distribution of OTFFC > FFC > TC among the three groups (*p* < 0.001 for all group comparisons). For the external rotation torque, the TC group is significantly greater than the FFC group and the OTFFC group (*p* = 0.001). For the ankle joint and subtalar joint, the plantar flexion torque of the ankle joint shows that the OTFFC group is significantly smaller than the FFC group and the TC group (*p* < 0.001). For the abduction torque of the subtalar joint, the OTFFC group is significantly higher than the FFC group and the TC group (*p* < 0.001).

**Table 2:** Effects of three left-foot gliding methods on lower limb joint torques during the gliding phase of the delivery action (*n* = 16) (Nm/kg).

Measure (moment)	FFC	OTFFC	TC	*F*	*p*	*η* ^2^
Hip joint flexion (+)/extension (−)	0.80 ± 0.08	0.85 ± 0.09	0.82 ± 0.07	1.604	0.211	0.06
Hip joint abduction (−)/ adduction (+)	0.17 ± 0.10^bc^	0.32 ± 0.07^a^	−0.05 ± 0.18^a^	38.762	0.001	0.60
Hip joint internal rotation (+)/external rotation (−)	2.13 ± 0.08^bc^	2.34 ± 0.09^ac^	2.20 ± 0.10^ab^	26.942	0.01	0.51
Knee joint flexion (−)/extension (+)	−0.24 ± 0.26^c^	−0.28 ± 0.34^c^	0.04 ± 0.14^ab^	8.23	0.01	0.24
Knee joint adduction (−)/ abduction (+)	−1.99 ± 0.09^bc^	−2.14 ± 0.11^ac^	−1.84 ± 0.13^ab^	34.724	<0.01	0.58
Knee joint internal rotation (+)/external rotation (−)	−0.84 ± 0.14^c^	−0.82 ± 0.10^c^	−1.12 ± 0.05^ab^	45.261	<0.01	0.64
Dorsiflexion (+)/plantarflexion (−)	−1.20 ± 0.29^b^	−0.65 ± 0.18^ac^	−1.29 ± 0.10^b^	49.476	<0.01	0.66
Subtalar joint abduction (−)/adduction (+)	−1.02 ± 0.16^b^	−1.34 ± 0.04^ac^	−0.93 ± 0.21^b^	34.685	<0.01	0.57

### 3.3 Muscle synergy characteristics

**Figure 6:**
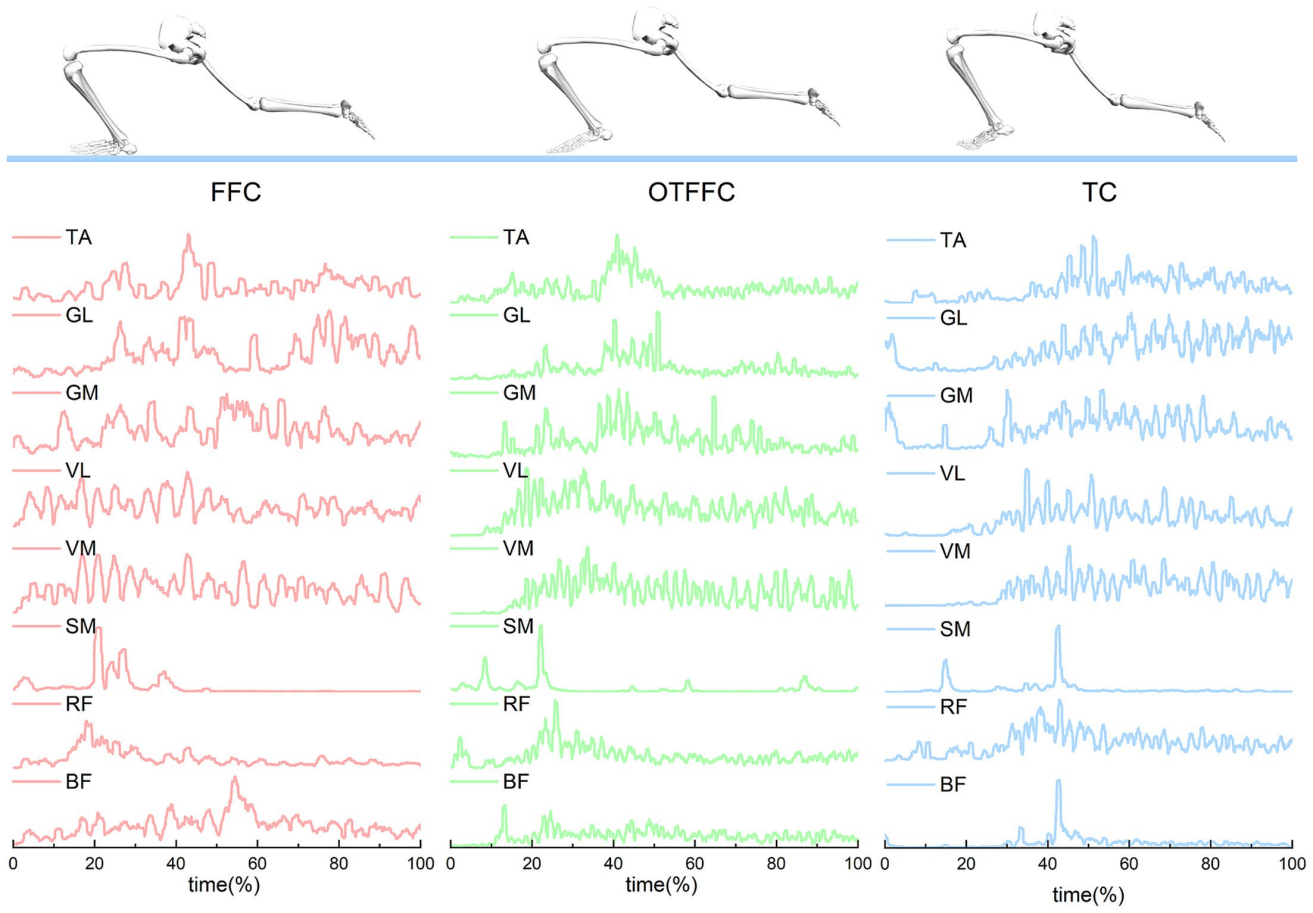
Muscle activity of the left lower limb during ice sliding in athletes using full-foot contact, out-toed full-foot contact, and toe contact techniques. This section presents the muscle activity of the left lower limb of the athletes, including the rectus femoris (RF), vastus medialis (VM), vastus lateralis (VL), semimembranosus (SM), biceps femoris (BF), medial gastrocnemius (GM), lateral gastrocnemius (GL), and tibialis anterior (TA), after preprocessing, during the three different curling sliding techniques.

Figure 6 illustrates that muscle activation curves are similar across the three sliding techniques, but each technique has its unique characteristics. Under the FFC condition, muscle activation remains relatively stable with minimal fluctuation, likely due to the even distribution of body weight and the high demand for stable muscle force output. In the OTFFC condition, certain muscles, such as the GL and VL, exhibit more pronounced activation peaks, possibly because these muscles need to generate greater contraction forces to maintain balance. For the TC, the activation of the TA and BF is significantly enhanced at specific time points. These specific characteristics reflect the differences in lower limb muscle recruitment patterns among the various sliding techniques.

#### 3.3.1 Synergy 1

As shown in Table 3, at least three muscle synergies can be extracted from the lower limb muscles under the three different gliding methods. There were no significant differences in the number of muscle synergies and the variance accounted for (VAF) among the three groups (*p* > 0.05). Synergy 1.

**Table 3:** Number of non-negative matrix factorization (NMF) muscle synergies for lower limb muscles under three gliding methods (*n* = 16).

Synergies & VAF (%)	FCC	OTFCC	TC	F	P	η2
Minimum number of synergies	3.03 ± 0.58	3.24 ± 0.37	3.17 ± 0.46	1.01	0.404	0.37
VAF (%)	97 ± 0.43	98 ± 0.67	99 ± 0.13	2.43	0.074	0.28

**Figure 7:**
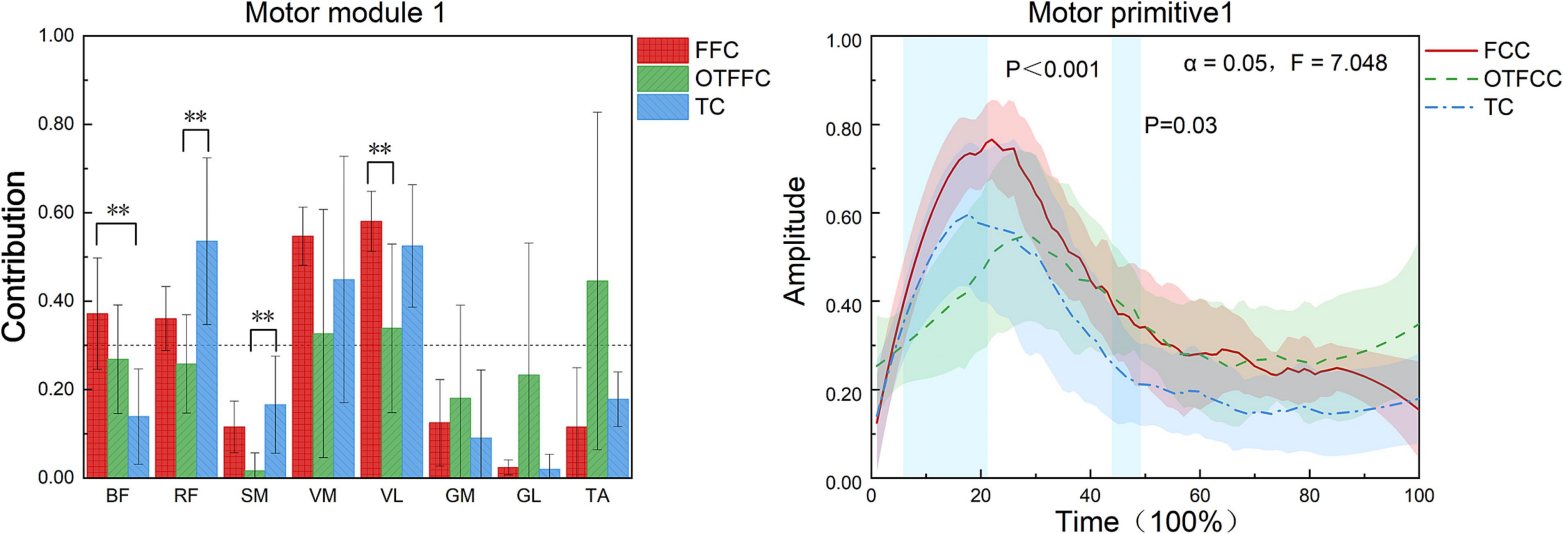
Effects of three gliding methods on the movement patterns and movement primitives of synergy 1 in the left lower limb muscles during the gliding phase of the delivery action (*n* = 16). ** indicates *p* < 0.01. The blue rectangular area in the right figure represents the interval with significant differences identified by SPM1d. The same applies to the following figures.

As shown in Figure 7, this study compared the muscle synergy characteristics of the lower limb during the gliding phase of curling delivery under different contact conditions and found significant differences in the muscle recruitment patterns of Synergy 1 among the three movement patterns (FFC, OTFFC, and TC). Specifically: In the FFC movement, Synergy 1 mainly recruits the BF, RF, and VM/VL. In the OTFFC movement, the primary muscles recruited are VM, VL, and TA. In the TC movement, the main muscles activated are RF, VM, and VL. Different contact conditions significantly affect the muscle contribution values of Synergy 1: the contribution value of BF shows significant inter-group differences (*F* = 6.022, *p* = 0.012), with the FFC group being significantly higher than the TC group (*p* = 0.01). The contribution value of RF also shows significant differences (*F* = 4.496, *p* = 0.03, η^2^ = 0.375), with the TC group being significantly higher than the OTFFC group (*p* = 0.01). The contribution value of SM changes significantly (*F* = 3.913, *p* = 0.043), with the TC group being significantly higher than the OTFFC group (*p* = 0.023). The contribution value of VL shows significant differences (*F* = 5.12, *p* = 0.02), with the FFC group being significantly higher than the OTFFC group (*p* = 0.024). The analysis of the movement primitive curves indicates significant differences in the activation patterns of Synergy 1 under different contact conditions during the 6–22% and 44–49% intervals of the movement cycle (*α* = 0.05, *F* = 7.048). This suggests that different gliding methods have specific effects on the temporal characteristics of movement control.

#### 3.3.2 Synergy 2

**Figure 8:**
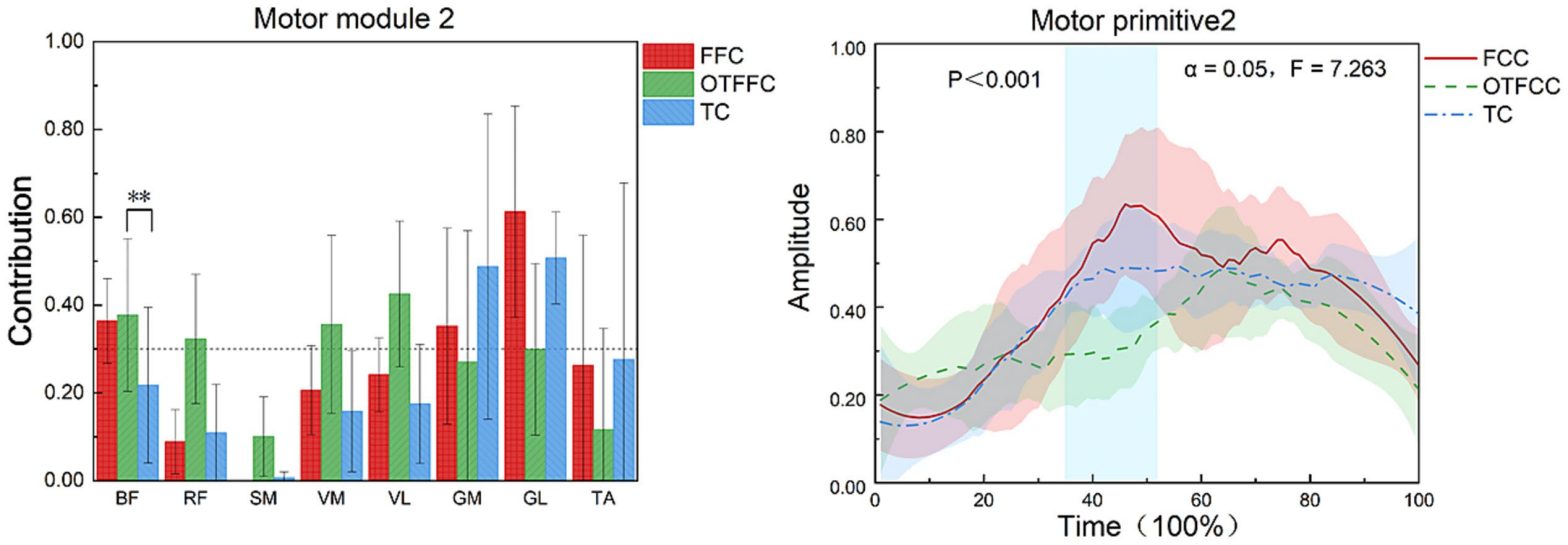
Effects of three gliding methods on the movement patterns and movement primitives of Synergy 2 in the left lower limb muscles during the gliding phase of the delivery action (*n* = 16). ** indicates *p* < 0.01. The blue rectangular area in the right figure represents the interval with significant differences identified by SPM1d.

As shown in Figure 8, in the FFC curling gliding action, Synergy 2 mainly involves the BF, GM, and GL. In the OTFFC curling gliding action, Synergy 2 mainly involves the BF, RF, VM, and VL. In the TC curling gliding action, Synergy 2 mainly involves the GM and GL. Different gliding methods significantly affect the contribution value of BF in Synergy 2 of the lower limb muscles (*F* = 22.165, *p* = 0.001). Specifically, there are significant differences in the contribution weights of BF among the FFC, OTFFC, and TC groups in Synergy 2 (*p* = 0.001). The OTFFC group has a significantly higher contribution weight than the FFC group, and the FFC group has a significantly higher contribution weight than the TC group. Different gliding methods also significantly affect the movement primitive curve changes in Synergy 2. During the 35–52% interval of the movement, there are significant differences in the movement primitive curve changes (*α* = 0.05, *F* = 7.263).

#### 3.3.3 Synergy 3

**Figure 9:**
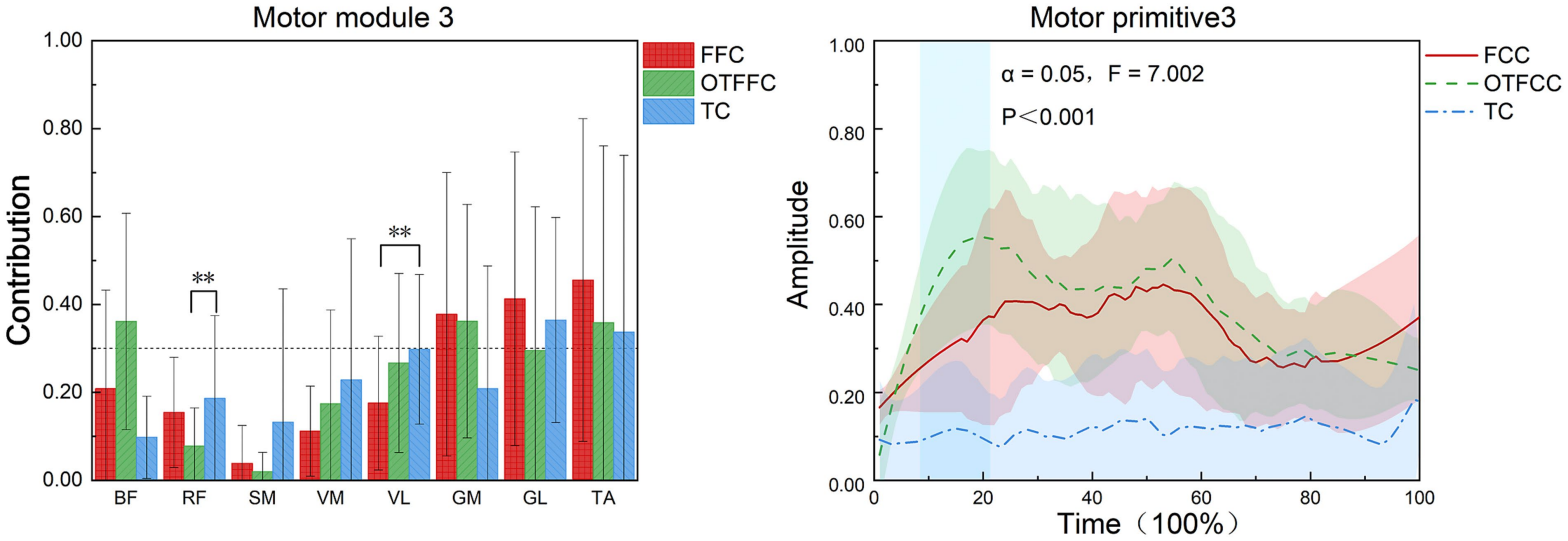
Effects of three gliding methods on the movement patterns and movement primitives of Synergy 3 in the left lower limb muscles during the gliding phase of the delivery action (*n* = 16). ** indicates *p* < 0.01. The blue rectangular area in the right figure represents the interval with significant differences identified by SPM1d.

As shown in Figure 9, in the FFC curling gliding action, Synergy 3 mainly involves the GM, GL, and TA. In the OTFCC curling gliding action, Synergy 3 also mainly involves GM, GL, and TA. In the TC curling gliding action, Synergy 3 mainly involves the participation of the GL and TA.

Different gliding methods significantly affect the contribution value of RF in Synergy 3 of the lower limb muscles (*F* = 4.975, *p* = 0.022, η^2^ = 0.399). In Synergy 3, the contribution weight of RF in the OTFFC group is significantly higher than that in the TC group (*p* = 0.031). They also significantly affect the contribution value of VL in Synergy 3 (*F* = 4.549, *p* = 0.029, η^2^ = 0.378), with the contribution weight of VL in the FFC group being significantly lower than that in the TC group (*p* = 0.03). Different gliding methods significantly affect the movement primitive curve changes in Synergy 3. During the 7–22% interval of the movement, there are significant differences in the movement primitive curve changes (α = 0.05, *F* = 7.002).

## 4 Discussion

The core finding of this study is the revelation that different curling propulsion techniques—FFC, OTFFC, and TC—correspond to distinct muscle synergy strategies. The TC technique, characterized by larger knee flexion amplitude and complex hip joint movement patterns, is closely associated with the significant enhancement of RF contribution and unique adjustments in BF contribution within its muscle synergy. Similarly, changes in the participation degree of VL observed in the OTFFC technique reflect the specialized synergy formed to adapt to the specific medial-lateral mechanical environment and force requirements of the knee joint.

### 4.1 Effects of gliding methods on joint kinematics

Previous research has shown that excessive flexion of the lower limb joints may lead to decreased joint stability, restricted movement function, and increased pain (Huang C. et al., 2023; Yang and Li, 2024). The results of this study indicate that during the curling delivery action, the hip joint is in a state of flexion, abduction, and external rotation, the knee joint is in a state of flexion, abduction, and internal rotation, and the ankle joint is in a state of dorsiflexion, which is consistent with previous studies (Yoo et al., 2012). The statistical analysis of this study highlights the TC gliding method as particularly noteworthy. The hip joint angle results show that the abduction angle during the TC gliding action is significantly greater than that of FFC and OTFFC, and the external rotation angle is significantly greater than that of FFC. This suggests that under the TC gliding method, athletes need to adjust their body center of gravity and lower limb support status through a larger hip abduction angle to maintain balance and control posture, thereby increasing the instability of the center of gravity during gliding and imposing higher demands on posture control. Additionally, the larger abduction angle may increase the stretching of the ligaments and soft tissues around the hip joint, thereby increasing the risk of injury (Markström et al., 2019; Glaviano and Bazett-Jones, 2021).

The results for the knee joint angles indicate that the flexion and internal rotation angles during TC are significantly higher than those during OTFFC and FFC. In contrast, the adduction angle of the knee joint during OTFFC is larger than that during FFC and TC. A greater knee flexion angle may suggest a larger range of joint motion and increased load-bearing capacity, which is influenced by factors such as muscle strength, joint flexibility, and movement technique (Lorenzetti et al., 2012). Additionally, the internal rotation angle of the knee joint during TC is significantly higher than that during OTFFC and FFC, while OTFFC demonstrates an advantage in the knee joint’s adduction angle. Differences in knee internal rotation and adduction angles may impact lower limb stability and movement control. For instance, a larger internal rotation angle can enhance the stability of the outer lower limb, whereas a larger adduction angle is advantageous for the transmission and control of force in the inner lower limb (Adouni and Shirazi-Adl, 2014).

The results for the ankle joint angles reveal that the dorsiflexion angle during OTFFC is significantly greater than that during TC. An increased ankle dorsiflexion angle allows the metatarsophalangeal joint to extend more fully, increasing the contact area between the foot and the ice surface, thereby improving propulsion efficiency and enhancing propulsive force. Ankle movement plays a critical role in maintaining body balance and controlling the direction of gliding. Different ankle angles can influence the athlete’s balance ability and the precision of gliding direction control.

The results for the subtalar joint angle showed that the abduction angle of the subtalar joint during TC propulsion was significantly smaller than during OTFFC. A larger subtalar joint abduction angle may allow the foot to better adapt to the unevenness and variability of the ice surface, thereby enhancing the athlete’s stability and safety. The movement of the subtalar joint is influenced by the lower limb muscles and the nervous system, and differences in abduction angles may reflect the athletes’ movement control and coordination abilities under different gliding methods. The findings of this study can serve as a reference for the training of curling athletes and the prevention of sports injuries.

### 4.2 Effects of gliding methods on lower limb joint torques

Different gliding methods significantly affect the torque changes in the three major joints (hip, knee, and ankle), potentially influencing the stability of the lower limb joints and the overall performance of athletes. In the results for the hip joint, the adduction torque exhibits a gradient distribution of “OTFFC group > FFC group > TC group,” while the internal rotation torque follows the pattern “OTFFC group > TC group > FFC group.” These differences may be closely related to lateral stability during gliding. A larger adduction torque helps maintain stability in the sagittal plane of the lower limbs, preventing excessive abduction or adduction, thereby effectively controlling the gliding posture (Glaviano and Bazett-Jones, 2021).

In the analysis of knee joint torques, the TC group demonstrates significantly higher flexion-extension torque compared to the OTFFC group, consistent with the findings of Iona Robertson et al. Compared to previous studies, we have included additional results on the adduction torque and external rotation torque of the knee joint. The results reveal a step-like distribution of “OTFFC > FFC > TC” for adduction torque, while the external rotation torque is significantly higher in the TC group than in the other two groups. Higher flexion-extension torque may indicate that the TC group can better control knee flexion and extension, thereby improving gliding efficiency and stability (Wang, 2022). However, the higher external rotation torque in the TC group may impose a greater burden on the lateral muscle groups of the knee joint, potentially increasing the risk of injuries such as meniscus damage (Shiwaku et al., 2022).

The torque analysis of the ankle joint reveals that the plantar flexion torque in the OTFFC group is significantly lower than that in the FFC and TC groups. This may result in reduced propulsive force during gliding in the OTFFC group, thereby affecting gliding speed and distance. Additionally, the subtalar joint abduction torque is significantly higher in the OTFFC group compared to the FFC and TC groups, potentially reflecting differences in energy transfer efficiency among the gliding methods.

From the perspective of sports injury risk assessment, joints subjected to high torques (particularly the knee and ankle joints) may face an increased risk of injury. The significant increase in knee flexion-extension torque and external rotation torque in the TC group may elevate the likelihood of knee injuries, such as meniscus damage and ligament strains. Therefore, achieving reasonable torque distribution and maintaining proper posture control are crucial for reducing the risk of sports injuries during gliding. By analyzing the torque distribution characteristics of different gliding methods, more targeted strategies for injury prevention can be developed for athletes, such as adjusting gliding postures or enhancing the training of specific muscle groups (Senter and Hame, 2006).

### 4.3 Effects of gliding methods on lower limb muscle synergy patterns

The muscle activation patterns in Synergy 1 exhibit distinct characteristics across different gliding techniques. The FFC gliding method primarily recruits the BF, RF, and VM/VL to stabilize lower limb joints and generate propulsive forces (Muyor et al., 2020). In the OTFFC propulsion technique, the VM, VL, and TA are predominant. Compared to TC, the kinematic characteristics of OTFFC involve greater ankle dorsiflexion and knee adduction. The dominant role of TA in synergy element 1 is to actively control and maintain a larger ankle dorsiflexion, thereby effectively adjusting the body’s center of mass. The combined participation of VM and VL provides dynamic stability to the knee joint during the adducted knee posture (Day et al., 2013). The TC propulsion technique primarily activates the RF, vastus medialis VM, and VL to meet its unique mechanical demands. There were significant differences in muscle contribution values between groups. The RF contribution value in the TC group was significantly higher than that in the OTFFC group. The SM contribution value was significantly higher in the TC group than in the OTFFC group, and the VL contribution value was significantly higher in the FFC group than in the OTFFC group. This synergistic pattern is closely related to the complex posture of TC, which involves large hip abduction and external rotation, as well as deep knee flexion with internal rotation. The hip flexion function of RF may be crucial for actively driving and maintaining the large hip abduction and external rotation posture after the significant lowering of the body’s center of mass in TC. The stabilization of the highly flexed knee joint and the coordinated activity with the semimembranosus to finely control the lower limb posture play a decisive role (Ramos et al., 2021). The analysis of movement primitive curves revealed significant differences in the activation patterns of Synergy 1 under different contact conditions during the 6–22% and 44–49% intervals of the movement cycle. These findings suggest that different gliding methods impose varying demands on muscle synergy activation patterns during key movement phases, such as the start, acceleration, and braking of gliding.

In Synergy 2, the FFC gliding method primarily involves the BF, GM, and GL, which are associated with knee joint stability and force production in the posterior lower leg. The OTFFC gliding method incorporates the BF, RF, VM, and VL, contributing to knee flexion-extension, stability, and overall force production in the lower limb. The higher contribution of the BF, which acts as a knee external rotator and flexor, may be related to the greater knee adduction movement in OTFFC. It likely participates in balancing and controlling the knee movement trajectory and the medial-lateral load distribution under this adducted posture (Kutzner et al., 2013). On the other hand, the extensive co-activation pattern of the quadriceps and hamstrings is generally associated with increasing joint stiffness and enhancing movement stability (Chang et al., 2015). Significant differences in the movement primitive curves were observed during the 35–52% interval of the movement cycle. These findings suggest that different gliding methods impose varying demands on muscle synergy activation patterns during this phase, potentially related to key movement segments such as acceleration, posture transition, or preparation for braking. In the TC sliding technique, Synergy 2 primarily involves the GM and GL, which control ankle stability and calf muscle activation. The BF contributes the least among the three groups. Given that the TC technique involves the largest knee flexion angle, it suggests that TC does not rely solely on the continuous isometric contraction of the BF to control deep knee flexion. Instead, it likely depends on the strong stabilizing action of the RF, VM, and VL in Synergy 1. Moreover, the dominant role of GM and GL in Synergy 2 highlights the reliance on ankle plantarflexion burst power and precise ankle posture control during TC sliding.

In Synergy 3, the FCC and OTFCC sliding techniques involve the GM, GL, and TA. This indicates that during the sliding process, GM and GL jointly control ankle plantarflexion, while TA adjusts dorsiflexion to achieve balance support. This mechanism is crucial for maintaining the overall dynamic stability and force output of the lower limbs. Among these, the contribution weight of the RF in Synergy 3 for the OTFFC group is significantly higher than that of the TC group. Combined with the larger ankle dorsiflexion angle in the OTFFC technique, the higher involvement of RF in Synergy 3 may indirectly affect the Synergistic control of the calf and ankle through the overall tension of the quadriceps femoris, or it may play a role during the forward shift of the body’s center of mass. Under the TC sliding technique, Synergy 3 mainly involves the GL and TA to meet its specific ankle and knee joint mechanical demands. Its contribution weight of the VL is significantly higher than that of the FCC group. In the TC sliding technique, a large knee flexion and internal rotation posture is required. The increased contribution of VL may counteract potential excessive internal rotation or eversion tendencies, and work in coordination with GL and TA to achieve fine-tuning of the ankle joint, meeting the specific mechanical demands of the ankle joint, which requires both flexibility for adjustment and stability for force transmission (Babadi et al., 2018). Additionally, the significant differences in the motor primitive curves in the early phase of the movement (7–22%) indicate that different sliding techniques have distinct muscle activation plans from the initial stage, which are directly related to the initial lower limb joint angle settings and early mechanical demands.

The muscle synergy patterns observed in curling athletes under different sliding techniques in this study may reflect not only differences in muscle recruitment strategies but also neural adaptations resulting from long-term specialized training. After thousands of repetitions in practice, the athletes’ nervous system organizes complex muscle activities into several functional synergistic modules. This simplifies the control process and enhances movement efficiency and stability (Yarrow et al., 2009; Singh et al., 2018). Therefore, the distinct core muscle combinations and their contribution weights exhibited under the TC, OTFFC, and FFC sliding techniques can be considered specialized neural control signatures formed by athletes to meet the unique biomechanical demands of each technique. This highlights the high plasticity and adaptability of the nervous system in motor control during motor skill learning.

## 5 Conclusion

Different delivery techniques in curling significantly impact the biomechanical characteristics of lower limb joints and muscle synergy patterns of the sliding leg. Specifically: TC technique: Shows greater hip abduction and external rotation, knee flexion and internal rotation, and subtalar joint abduction compared to OTFFC and FFC, indicating higher demands on hip and knee range of motion and distinct subtalar joint biomechanics. OTFFC technique: Exhibits larger knee adduction and ankle dorsiflexion angles than TC and FFC, emphasizing unique requirements for these joints. Muscle synergy patterns: In Synergy 1, the rectus femoris contributes more in TC than in OTFFC, while the VL contributes less in OTFFC than in FFC. The biceps femoris contributes less in TC than in FFC. In Synergy 2, BF contribution is lower in TC than in OTFFC.

These biomechanical differences suggest that TC relies more on RF-involved muscle synergies, while OTFFC involves reduced VL participation. Athletes should strengthen specific muscle groups based on their technique: hip abductors, knee flexors/extensors, and external rotators for TC; ankle dorsiflexors and subtalar joint abductors for OTFFC. This targeted training enhances performance and reduces injury risk.

## Data Availability

The raw data supporting the conclusions of this article will be made available by the authors, without undue reservation.

## Footnotes

^1^ https://simtk.org/projects/full_body
